# Explainable Lightweight AI for the Identification of Right-Sided Cardiac Dysfunction in a Saudi Arabian Diabetic Cohort

**DOI:** 10.3390/jcm15124719

**Published:** 2026-06-17

**Authors:** Umar Hasan, Metab Algeffari, Haifa F. Alhasson, Muhammad Ali Nayeem

**Affiliations:** 1 Department of Electrical and Computer Engineering, School of Engineering and Physical Sciences, North South University, Dhaka 1229, Bangladesh; umar.hasan@northsouth.edu; 2 Department of Family and Community Medicine, College of Medicine, Qassim University, Buraydah 51452, Saudi Arabia; m.geffari@qu.edu.sa; 3 Abdullah Al-Othaim Diabetes Center, Medical City, Qassim University, Buraydah 52571, Saudi Arabia; 4 Department of Information Technology, College of Computer, Qassim University, Buraydah 51452, Saudi Arabia; hhson@qu.edu.sa; 5 Department of Computer Engineering, College of Computer, Qassim University, Buraydah 51452, Saudi Arabia

**Keywords:** tricuspid valve dysfunction, artificial intelligence, machine learning, echocardiography, clinical triage, explainable AI, Saudi Arabia, diabetes mellitus, risk stratification, digital health

## Abstract

**Background**: Tricuspid valve dysfunction is historically underdiagnosed, and right-sided cardiac abnormalities are clinically important in high-risk diabetic populations. Diabetes may promote right-sided dysfunction through cardiometabolic remodeling, diastolic dysfunction, and elevated pulmonary pressures. This study introduces an explainable, lightweight artificial intelligence framework to infer extreme right heart phenotypes from left-sided echocardiographic and systemic clinical markers. **Methods**: We retrospectively analyzed an existing clinical dataset of approximately 370 Saudi Arabian individuals with diabetes mellitus. Seven baseline machine learning classifiers were evaluated using leak-aware preprocessing. To reduce optimism bias, models were validated with a True Nested Cross-Validation protocol. Probabilistic calibration, parameter reduction, and computational efficiency were prioritized for clinical triage, and SHapley Additive exPlanations (SHAP) supported transparent decision-making. **Results**: Random Forest was selected for its balance of discrimination and calibration. Under True Nested Cross-Validation, it achieved an AUC-ROC of 0.8602, AUC-PR of 0.8343, accuracy of 0.8075, sensitivity of 0.7733, specificity of 0.8239, Brier Score of 0.1281, Calibration Slope of 1.1720, and Observed/Expected Ratio of 0.8522. Feature ablation indicated a holistic cardiometabolic severity signal, with left atrial dimensions, diastolic dysfunction grade, and diuretic use as primary predictors. The model required 205.00 kilobytes of storage and 14.1956 milliseconds for inference. **Conclusions**: Left-sided cardiac markers combined with systemic indicators can flag concurrent severe right-sided abnormalities. This lightweight framework is a promising triage-oriented screening prototype for prioritizing echocardiographic assessment in high-risk diabetic patients during routine clinical visits without requiring specialized hardware; however, until prospective external validation is completed, this study must strictly be viewed as hypothesis-generating.

## 1. Introduction

Tricuspid regurgitation has long been labeled the “forgotten valve” in clinical cardiology and is receiving increasing attention as a potentially important public health concern [1]. While significant clinical focus is traditionally directed toward left-sided cardiac structures, tricuspid valve dysfunction (TVD) is now recognized as an independent contributor to morbidity and mortality [2,3]. Prevalence studies indicate that moderate to severe TVD affects nearly 4 percent of individuals over the age of 75, yet many of these patients remain untreated, in part due to the historical underappreciation of the disease [1,4]. Secondary tricuspid regurgitation is the most frequent etiology of tricuspid valve pathology in Western cohorts and has historically been undertreated because of the misconception that it will resolve after correction of the primary left-sided lesion [5]. The pathophysiology of right heart dysfunction is frequently a secondary consequence of left heart disease (LHD), where chronic left-sided deterioration elevates pulmonary pressures and induces right ventricular strain [6,7,8]. This clinical cascade may be particularly relevant in patients with diabetes mellitus, a population characterized by metabolic disturbances that drive structural cardiac remodeling [9,10,11]. Diabetes is strongly associated with diastolic dysfunction and HFpEF, which may indirectly contribute to progressive right ventricular strain and tricuspid annular dilation [12,13,14,15,16]. Within Saudi Arabia and the broader Middle East, the high prevalence of diabetes and associated heart failure risk factors underscores the need for localized, data-driven strategies for early cardiovascular screening [17,18].

Despite its critical prognostic importance, tricuspid dysfunction remains notoriously difficult to diagnose due to the complex, retrosternal geometry of the right heart and the frequent absence of direct visualization on standard echocardiograms [19,20]. This often leads to late clinical referrals, where surgical intervention is associated with high in-hospital mortality rates [21,22]. Although artificial intelligence (AI) has demonstrated substantial potential in cardiovascular diagnostics, current computational frameworks have predominantly focused on left-sided pathologies or general heart failure phenotyping [23,24,25]. Furthermore, a significant knowledge gap persists between machine learning developers and clinical practitioners, primarily because many state-of-the-art models function as opaque black boxes requiring immense computational resources [26,27]. This lack of transparency and efficiency severely hampers the integration of AI into real-world digital therapeutics and clinical decision support systems, where interpretability, low-parameter performance, and deployment safety are paramount for trust and clinical use [28,29,30,31].

To address these diagnostic and technical limitations, we propose an explainable and lightweight machine learning framework designed to function as a triage-oriented screening prototype for identifying an extreme right heart phenotype. Our approach leverages left-sided echocardiographic and concurrent systemic clinical markers from a Saudi Arabian diabetic cohort to infer right-sided status, reducing reliance on direct tricuspid visualization. This broader systems-oriented strategy is consistent with recent data-driven clinical modeling studies showing that routinely collected systemic indicators can support meaningful risk stratification beyond a single organ-centric view [32]. Unlike traditional high-parameter models, our system prioritizes computational efficiency and rigorous probabilistic calibration to support potential suitability for clinical integration [33,34,35]. By integrating explainability techniques, we present a more transparent tool that may align with the needs of modern geriatric and cardiovascular care [36,37]. The key contributions of this study are as follows:

- We developed a diagnostic triage pipeline that identifies severe right heart abnormalities by utilizing concurrent left-sided cardiac deterioration markers, with the aim of helping address the relative underassessment of the right heart.
- We validated the predictive framework within a geographically specific clinical cohort of Saudi Arabian patients with diabetes mellitus, providing regional epidemiological relevance.
- We implemented a True Nested Cross-Validation protocol to demonstrate that lightweight machine learning models can achieve strong, unbiased diagnostic accuracy and probabilistic calibration while maintaining low computational complexity.
- We employed SHapley Additive exPlanations (SHAP) [38] to improve interpretability of the model predictions and conducted rigorous feature ablation to confirm the model captures a shared cardiometabolic severity signal.

The remainder of this article is organized as follows. Section 2 reviews the relevant literature on artificial intelligence in echocardiography, diabetic cardiovascular manifestations, and right heart assessment. Section 3 describes the study cohort, preprocessing pipeline, model development strategy, and explainability framework. Section 4 presents the experimental findings and comparative performance analysis, while Section 5 discusses the clinical implications, interpretability insights, limitations, and future research directions. Finally, Section 6 concludes the article by summarizing the principal contributions and translational significance of the proposed framework.

## 2. Related Work

The convergence of artificial intelligence and cardiovascular medicine has facilitated a paradigm shift in the management of complex cardiac pathologies, particularly as heart failure continues to impose a major clinical burden across populations. While substantial research has addressed left-sided heart disease and general diabetic complications, the computational prediction of right-sided abnormalities remains a neglected frontier. Table 1 provides a comprehensive summary of the foundational literature and state-of-the-art computational frameworks that contextualize this study.

### 2.1. Artificial Intelligence in Echocardiography

Machine learning and deep learning have revolutionized the interpretation of echocardiographic data, moving toward what Topol [40] described as high-performance medicine. Modern algorithms now automate the quantification of ejection fraction and the identification of structural abnormalities with accuracy comparable to human experts [39]. Recent advancements have also explored the use of large language models, wearable and remote-monitoring systems, electrocardiographic deep learning, multimodal visualization, and AI-enabled trial workflows to streamline cardiovascular prediction and adjudication [26,41,42,43,44]. Furthermore, explainable artificial intelligence (XAI) frameworks, such as XAI-HD, have been proposed to enhance clinician trust by providing transparent feature importance analysis for heart disease detection [25]. Despite these successes, most current models rely on highly parameterized architectures that require substantial computational power, which limits their utility in resource-constrained environments or portable digital therapeutics [27,28]. There is an urgent need for lightweight models that maintain high diagnostic precision while minimizing parameter counts, particularly for real-time clinical decision support [29,34].

### 2.2. Cardiac Manifestations in Diabetes Mellitus

Patients with type 2 diabetes face an elevated risk of cardiovascular disease, which remains the leading cause of mortality in this demographic [10]. The pathophysiology of diabetic cardiomyopathy involves metabolic disturbances that drive left ventricular hypertrophy and diastolic dysfunction independently of coronary artery disease [9,45]. Regional studies in the Middle East have highlighted the high burden of heart failure with preserved ejection fraction (HFpEF) and associated comorbidities such as hypertension and dyslipidemia in diabetic cohorts [11,17]. Computational efforts have targeted these left-sided complications, with models developed to predict left ventricular diastolic dysfunction (LVDD), incident heart failure hospitalization, broader cardiovascular subtypes, and phenotype treatment responses in HFpEF patients [23,24,46]. However, these frameworks often ignore the secondary impact of left-sided deterioration on the right heart. Our work builds upon these findings by suggesting that left-sided markers and systemic indicators, such as those analyzed in diabetic risk stratification [35], may be leveraged to detect concurrent right-sided dysfunction.

### 2.3. Right Heart Assessment and the “Forgotten Valve”

The tricuspid valve has historically received less attention in clinical monitoring, a pattern associated with delayed diagnosis and high surgical risk in advanced disease [1,21]. Severe tricuspid regurgitation (TR) is an independent predictor of poor survival regardless of left ventricular function or pulmonary pressures [2,47]. The management of TR is particularly complex because right-sided structures are often difficult to visualize on standard transthoracic echocardiograms, frequently leading to underdiagnosis until the disease reaches an advanced stage [19,20]. Recent consensus statements have emphasized the need for early identification of right heart failure, particularly when it is secondary to left-sided valvular or metabolic disease [4,47]. While some studies have explored the impact of TR on hemodynamics during exercise or in patients with cardiac implants, automated triage tools that do not rely on direct tricuspid visualization remain limited. By proposing a lightweight, inferential AI framework, this study aims to contribute to the development of an “automated second opinion” for identifying patients who may warrant closer right-heart assessment [36,37].

## 3. Methods

**Figure 1:**
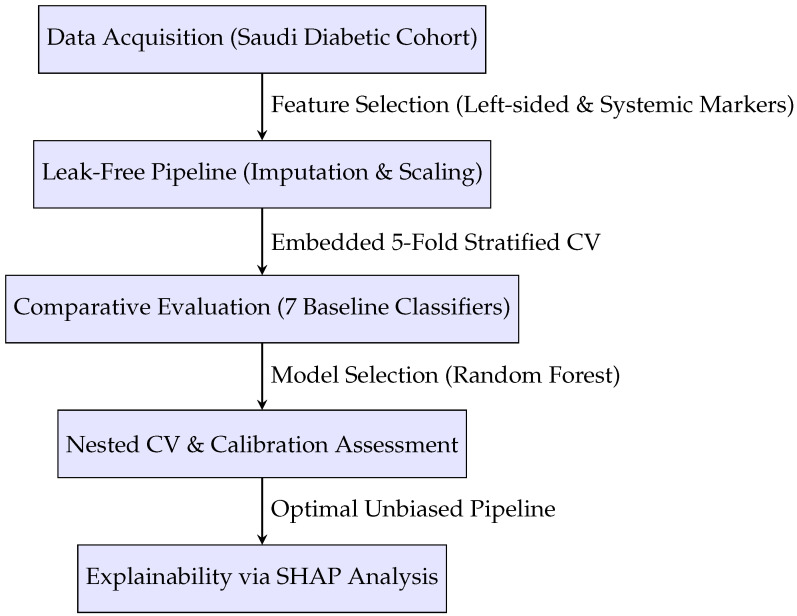
Flowchart illustrating the systematic methodology for predicting the extreme right heart phenotype using an explainable, lightweight, and leak-free artificial intelligence pipeline.

The analytical framework developed for this study encompasses data acquisition, rigorous preprocessing, the training of lightweight machine learning models, ensemble optimization, and transparent model evaluation. Figure 1 illustrates the comprehensive architecture of the proposed clinical decision support system.

### 3.1. Study Design and Reporting Guidelines

To ensure methodological rigor, transparency, and reproducibility in the development of clinical predictive models, this retrospective diagnostic accuracy study was conducted and reported in strict adherence to the TRIPOD+AI (Transparent Reporting of a multivariable prediction model for Individual Prognosis Or Diagnosis + Artificial Intelligence) statement and the original TRIPOD reporting principles [48]. Furthermore, given the explicit diagnostic and triage claims of the framework, the evaluation and reporting protocols were simultaneously aligned with the STARD-AI (Standards for Reporting of Diagnostic Accuracy Studies + Artificial Intelligence) guidelines [49].

### 3.2. Clinical Endpoint Definition and Severity Grading

To ensure clinical precision and address the diagnostic ambiguity of isolated right-sided markers, the primary predictive endpoint was rigorously defined as an “Extreme Right Heart Phenotype.” The ground truth labels were derived from standard guideline-based echocardiographic assessments provided in the source dataset.

To formulate a robust and clinically unambiguous binary diagnostic target, patients were classified into two distinct cohorts:

1. Normal Right Heart (Class 0): Patients exhibiting strictly normal Tricuspid Valve Regurgitation (TVR grade 1) and a structurally normal Right Ventricular size (RV grade 1).
2. Severe Right Heart Abnormality (Class 1): Patients exhibiting moderate to severe Tricuspid Valve Regurgitation (TVR grade ≥ 3) or clinically significant Right Ventricular dilation (RV grade ≥ 2).

Patients falling into indeterminate or mild boundary zones (e.g., isolated mild regurgitation without dilation) were strictly excluded from the training manifold to preserve the absolute integrity of the diagnostic labels. This composite endpoint ensures the model predicts a definitive, severe right-sided structural and functional decline rather than a marginal or ambiguous echocardiographic finding.

### 3.3. Problem Formulation

The clinical objective of identifying tricuspid valve abnormality is mathematically formulated as a binary classification task. Let $D={\left\{\left(x_{i},y_{i}\right)\right\}}_{i=1}^{N}$ represent the clinical dataset consisting of *N* patients. For each patient *i*, $x_{i}\in R^{d}$ denotes a *d*-dimensional feature vector comprising patient demographics, clinical comorbidities, and left-sided echocardiographic parameters. The target variable $y_{i}\in \left\{0,1\right\}$ represents the Extreme Right Heart Phenotype, where $y_{i}=0$ indicates a structurally and functionally normal right heart and $y_{i}=1$ denotes a severe right-sided abnormality.

The goal is to learn a predictive mapping function $f:R^{d}\to \left[0,1\right]$ parameterized by θ such that $f_{\theta }\left(x_{i}\right)={\hat{p}}_{i}$, where ${\hat{p}}_{i}$ is the predicted probability of the patient having a right-sided abnormality. The final class label ${\hat{y}}_{i}$ is assigned using a standard decision threshold of 0.5:

(1) $${\hat{y}}_{i}=\left\{\begin{array}{cc} 1, & \mathrm{if}\;{\hat{p}}_{i}\geq 0.5\\ 0, & \mathrm{otherwise} \end{array}\right.$$

### 3.4. Data

#### 3.4.1. Dataset

The dataset utilized in this study comprises a retrospective single-center cohort of N=367 Saudi Arabian patients diagnosed with diabetes mellitus. The clinical features incorporate comprehensive systemic measurements including age, sex, weight, body mass index, and blood pressure, alongside concurrent diagnoses such as atrial fibrillation, hypertension, and ischemic heart disease. Crucially, the dataset includes quantifiable left-sided echocardiographic markers such as left atrium volume and left ventricular mass.

The Extreme Right Heart Phenotype was defined based on strictly graded echocardiographic assessments recorded in the source clinical dataset and used as the composite binary study endpoint for model development and evaluation.

**Figure 2:**
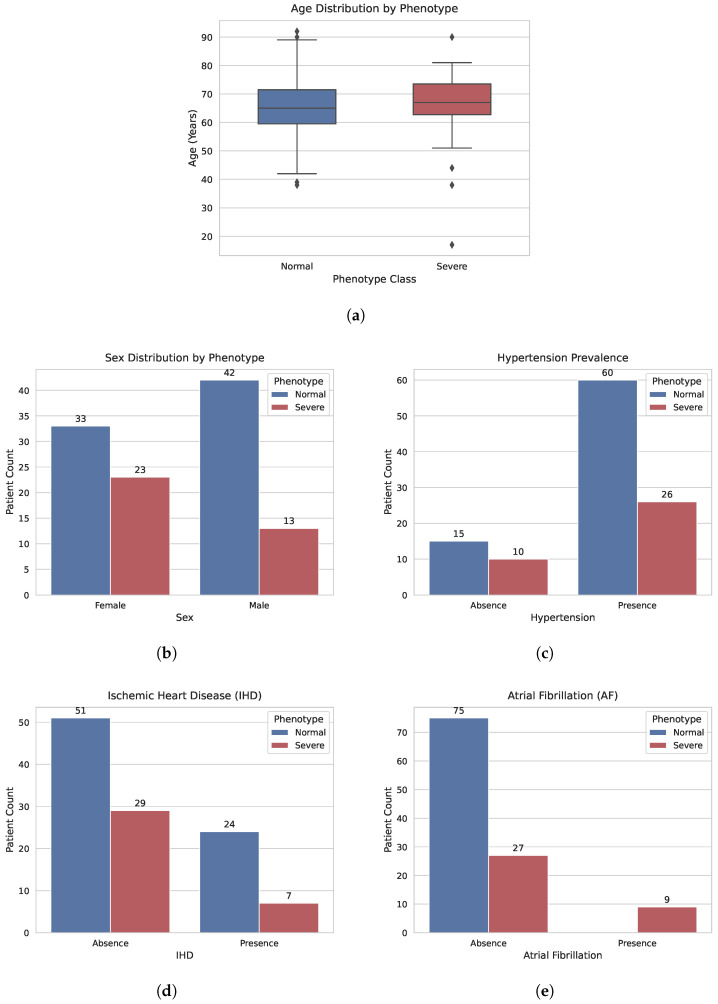
Baseline cohort characteristics stratified by the Extreme Right Heart Phenotype. (**a**) Age distribution of the patients. (**b**) Sex distribution within the cohort. (**c**) Prevalence of concurrent Hypertension. (**d**) Prevalence of Ischemic Heart Disease (IHD). (**e**) Prevalence of Atrial Fibrillation (AF).

Exploratory data analysis was conducted to establish the baseline patient characteristics and uncover interdependencies between systemic metabolic indicators and cardiac structural changes. Figure 2 presents a summary of the cohort demographics and the prevalence of key cardiovascular comorbidities stratified by the extreme right heart phenotype. The visualizations confirm a high prevalence of concurrent conditions, particularly atrial fibrillation and hypertension, underscoring the complex clinical presentation of the patient population.

**Figure 3:**
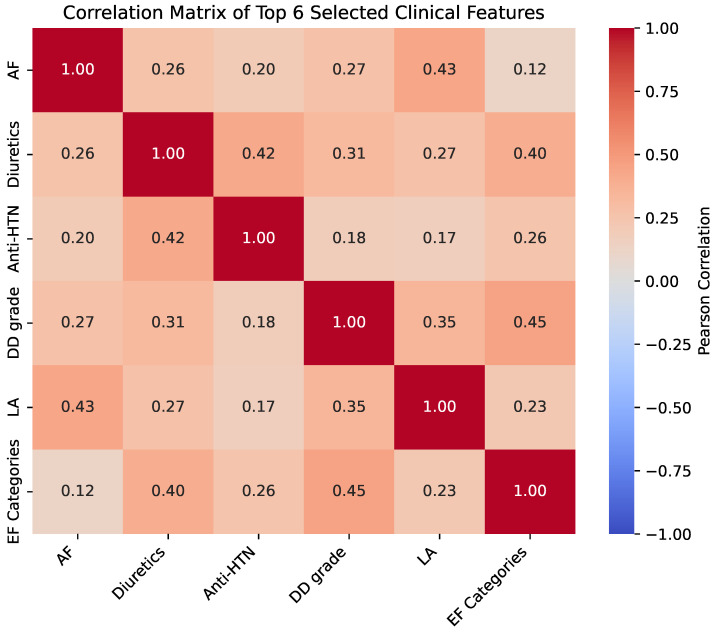
Pearson correlation matrix illustrating the linear interdependencies among the six most influential clinical and echocardiographic features identified by the predictive pipeline.

To quantitatively evaluate the linear interdependencies among the most influential clinical and echocardiographic features identified by our predictive pipeline, a Pearson correlation matrix was generated (Figure 3). This analysis provides a transparent view of the relationships between structural markers (such as Left Atrial volume and Diastolic Dysfunction grade), electrical indicators (Atrial Fibrillation), and pharmacological management (Diuretics and Anti-Hypertensive therapy), offering clinical context prior to the final predictive modeling.

**Figure 4:**
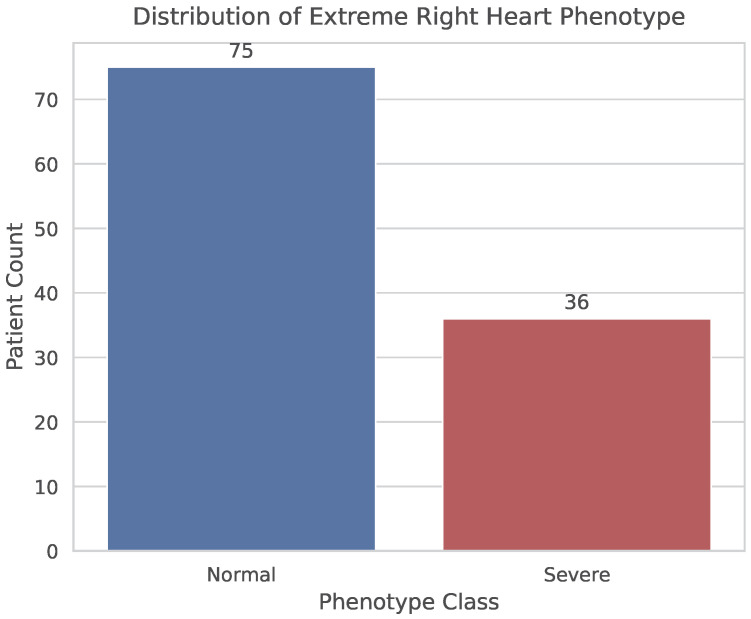
Distribution of the primary target variable (Extreme Right Heart Phenotype) indicating normal (0) and severe (1) classifications within the study population.

Furthermore, Figure 4 displays the distribution of the primary target variable, the Extreme Right Heart Phenotype, explicitly detailing the class prevalence (75 Normal versus 36 Severe) within the study population.

To ensure clarity regarding the input variables, the predictive pipeline ingested the complete 48-variable feature space summarized by the exploratory analysis in Figure 3. The echocardiographic component includes critical structural and functional measurements, specifically Ejection Fraction Categories (EF Categories), Left Ventricular Mass Index (LV mass2), Interventricular Septum Thickness (IVS Thick), and Diastolic Dysfunction Grade (DD grade). To capture both volumetric and linear atrial remodeling, both Left Atrial Volume (LA) and anteroposterior Left Atrial Dimension (LA dimension) are evaluated as distinct variables. Table 2 summarizes the primary clinical categories and the most highly ranked variables represented within this broader predictive feature space.

#### 3.4.2. Preprocessing

To reduce the risk of data leakage, direct patient identifiers (specifically the DN column) were removed from the feature space prior to any analytical steps. Furthermore, direct target components and right-sided echocardiographic parameters (TVS, TVR, PVS, PVR, and RV size) were explicitly excluded from the feature space prior to modeling. Removing these variables was strictly required to prevent data leakage and tautological right-sided predictions, ensuring the model relied exclusively on left-sided and systemic markers to infer right heart status. However, Pleural Effusion was retained. The clinical rationale for its inclusion is that pleural effusion can serve as a systemic proxy for pulmonary congestion, potentially acting as a physiological bridge between left heart failure and subsequent right heart dysfunction.

To address class imbalance within the training manifold, the Synthetic Minority Over-sampling Technique (SMOTE, k=3) was utilized. Following imputation and scaling, automated feature selection was performed using Analysis of Variance (ANOVA) F-values to isolate the most discriminative predictors. To strictly prevent data leakage, both SMOTE and the ANOVA feature selection were embedded as transformation steps within the cross-validation pipelines, ensuring synthetic samples and feature scores were generated exclusively from the training folds and were never exposed to the test partitions.

Missing values within continuous and categorical features were addressed using median imputation, a pragmatic alternative to more elaborate chained-equation imputation when preserving a leak-free cross-validation pipeline is prioritized [50]. Let $x_{\cdot j}$ represent the *j*-th feature vector across all *N* patients. The imputed value for a missing entry $x_{ij}$ is defined as:

(2) $$x_{ij}^{\left(imp\right)}=\left\{\begin{array}{cc} x_{ij}, & \mathrm{if}\;x_{ij}\;\mathrm{is}\;\mathrm{observed}\\ \mathrm{median}(x_{\cdot j}), & \mathrm{if}\;x_{ij}\;\mathrm{is}\;\mathrm{missing} \end{array}\right.$$

Following imputation, standard scaling was applied to all features to ensure equal contribution to distance-based algorithms and to accelerate convergence during optimization. The normalized feature $z_{ij}$ is computed as:

(3) $$z_{ij}=\frac{x_{ij}^{\left(imp\right)}-\mu_{j}}{\sigma_{j}}$$

where $\mu_{j}$ and $\sigma_{j}$ are the mean and standard deviation of the *j*-th feature, respectively.

To guarantee a leak-free validation process, both the median imputation and standard scaling steps were deeply embedded within scikit-learn Pipeline objects during the cross-validation. This architecture ensures that preprocessing transformations were strictly fit on the training folds and subsequently applied to the test folds, completely eliminating any risk of data leakage.

### 3.5. Model Architecture and Nested Validation Framework

We evaluated seven lightweight machine learning algorithms to identify the optimal predictive architecture: Logistic Regression [51], K-Nearest Neighbors [52], Support Vector Machine [53], Naive Bayes, Multilayer Perceptron (MLP), Random Forest [54], and XGBoost [55]. Ensemble learning was considered particularly relevant because robust aggregation strategies have long been used to improve classifier stability, including recent SHAP-guided weighted voting designs in biomedical classification.

To completely eliminate the optimism bias inherent in standard k-fold model selection and threshold tuning, we implemented a True Nested Cross-Validation protocol for our final model evaluation. The outer loop consisted of a 5-fold Stratified Cross-Validation to assess unbiased generalization performance. Within each outer training fold, an inner 3-fold Stratified Cross-Validation (Grid Search) was executed to dynamically optimize hyperparameters and feature selection thresholds. This strict separation ensures that the performance metrics reported are not artificially inflated by exposing the test data to the optimization phase, consistent with rigorous internal-validation workflows used in other clinical machine learning studies. Algorithm 1 formalizes this rigorous validation and threshold optimization process.
**Algorithm 1** True Nested Cross-Validation and Threshold Optimization1:**Input:** Processed dataset D, model family *M*, hyperparameter space Θ, $K_{out}=5$, $K_{in}=3$2:**Output:** Unbiased out-of-fold probabilities $\hat{P}$, optimal threshold $\tau^{*}$3:Initialize empty array $\hat{P}$ of size |D|4:**for** each outer fold $k\in \{1,\ldots ,K_{out}\}$ **do**5:   Split D into $D_{train}$ and $D_{test}$ preserving class prevalence6:   *// Inner Loop: Hyperparameter Optimization*7:   Perform $K_{in}$-fold Grid Search on $D_{train}$ over space Θ8:   Select optimal hyperparameters $\theta^{*}$ maximizing inner cross-validation accuracy9:   *// Outer Loop: Unbiased Evaluation*10:   Train model $M_{\theta^{*}}$ on the entire $D_{train}$ partition11:   Compute predicted probabilities for $D_{test}$ and store in $\hat{P}$12:**end for**13:*// Global Threshold Optimization*14:Evaluate all candidate decision thresholds on unbiased probabilities $\hat{P}$15:Select optimal $\tau^{*}$ that maximizes min(Sensitivity, Specificity) subject to global Accuracy≥0.8016:**return**$\;\hat{P},\tau^{*}$

### 3.6. Evaluation Metrics

Model performance was rigorously quantified using a comprehensive suite of metrics appropriate for medical biomarker validation [56]. Let TP, TN, FP, and FN denote True Positives, True Negatives, False Positives, and False Negatives, respectively. The fundamental discrete metrics are defined as follows:

(4) $$\mathrm{Accuracy}=\frac{TP+TN}{TP+TN+FP+FN}$$

(5) $$\mathrm{Precision}\;\left(\mathrm{PPV}\right)=\frac{TP}{TP+FP}$$

(6) $$\mathrm{Recall}\;\left(\mathrm{Sensitivity}\right)=\frac{TP}{TP+FN}$$

(7) $$\mathrm{Specificity}=\frac{TN}{TN+FP}$$

(8) $$\mathrm{Negative}\;\mathrm{Predictive}\;\mathrm{Value}\;\left(\mathrm{NPV}\right)=\frac{TN}{TN+FN}$$

(9) $$F1\;\mathrm{Score}=2\times \frac{\mathrm{Precision}\times \mathrm{Recall}}{\mathrm{Precision}+\mathrm{Recall}}$$

To assess probabilistic reliability, we calculated the Brier Score, which measures the mean squared difference between predicted probabilities ${\hat{p}}_{i}$ and the actual outcomes $y_{i}$ [57]:

(10) $$\mathrm{Brier}\;\mathrm{Score}=\frac{1}{N}\sum_{i=1}^{N}{\left({\hat{p}}_{i}-y_{i}\right)}^{2}$$

In addition to the numerical Brier Score, probabilistic calibration was visually assessed using calibration curves. These curves map the mean predicted probabilities against the true fraction of positive outcomes to verify that the confidence of the model accurately reflects the actual clinical risk. Furthermore, the AUC ROC and the Area Under the Precision–Recall Curve (AUC PR) were computed to evaluate discriminatory capacity across all classification thresholds, with the AUC PR being particularly informative for clinical datasets [58,59]. Their continuous forms can be expressed as:

(11) $$\mathrm{AUC}\text{-}\mathrm{ROC}=\int_{0}^{1}\mathrm{TPR}\left({\mathrm{FPR}}^{-1}\left(u\right)\right)\;du=\int_{0}^{1}\mathrm{TPR}\left(t\right)\;d\mathrm{FPR}\left(t\right)$$

(12) $$\mathrm{AUC}\text{-}\mathrm{PR}=\int_{0}^{1}\mathrm{Precision}\left(r\right)\;dr$$

where TPR denotes the true positive rate, FPR the false positive rate, and *r* recall. To establish rigorous statistical confidence, all reported metrics include 95 percent Confidence Intervals (95% CI). To directly address the limitations of small cohort sizes, these intervals were derived by performing 1000 bootstrap iterations explicitly on the pooled out-of-fold predictions generated by the cross-validation framework, ensuring the intervals reflect true out-of-sample variance [60].

Furthermore, to satisfy the stringent requirements of clinical risk stratification tools, we expanded our probabilistic assessment beyond standard Brier Scores. We calculated advanced calibration metrics, including the Calibration Slope (ideal value = 1.0), the Calibration Intercept (ideal value = 0.0), and the Observed/Expected (O/E) Ratio (ideal value = 1.0). These were derived by fitting a logistic regression model to the logit-transformed predicted probabilities against the true clinical outcomes, allowing us to mathematically quantify risk overestimation or underestimation.

### 3.7. Model Explainability

To overcome the inherent opacity of complex machine learning algorithms, we integrated SHapley Additive exPlanations (SHAP) into our predictive pipeline. SHAP utilizes a game-theoretic approach to assign a precise importance value to each feature for every individual prediction, addressing the usability challenges common in clinical decision support systems [25,29,38].

Mathematically, let *F* represent the complete set of all input clinical and echocardiographic features. The Shapley value $\phi_{j}$ for a specific feature *j*, given a model prediction f(x), is calculated by evaluating the marginal contribution of feature *j* across all possible feature subsets S⊆F∖{j}:

(13) $$\phi_{j}=\sum_{S\subseteq F\setminus \{j\}}\frac{|S|!(|F|-|S|-1)!}{|F|!}\left[f_{x}\left(S\cup \left\{j\right\}\right)-f_{x}\left(S\right)\right]$$

where $f_{x}\left(S\right)$ denotes the expected model output conditioned exclusively on the subset of features *S*.

In this study, the SHAP explainability framework was applied strictly to the Random Forest model. Although XGBoost achieved a marginally higher Area Under the Receiver Operating Characteristic Curve (AUC-ROC), the Random Forest was deliberately selected for explainability analysis due to its superior Sensitivity (0.9884 versus 0.9768 for XGBoost). In the context of clinical triage for identifying severe valvular abnormalities, minimizing false negatives is the highest priority. Therefore, the Random Forest represents the optimal framework for detailed transparent evaluation. We generated global feature impacts across the entire Saudi Arabian diabetic cohort using SHAP Summary and Beeswarm plots. Standard Feature Importance plots ranked the clinical markers based on their mean absolute Shapley values. Furthermore, Waterfall and Decision plots elucidated the step-by-step logic behind local, patient-specific predictions, while Dependence plots revealed complex, non-linear interactions between critical left-sided cardiac parameters and the probability of right heart dysfunction.

### 3.8. Experimental Setup

To ensure complete reproducibility and reliable sample size analysis [61], a fixed random seed of 42 was applied globally across all computational processes, data splitting mechanisms, and model initializations. The models were validated utilizing a 5-fold Stratified Cross-Validation protocol to preserve the target class distribution across all training and testing partitions.

All computational experiments were executed within a Kaggle cloud environment utilizing standard CPU compute resources without any GPU hardware acceleration. The software stack was built upon Python 3.12.13, leveraging the scikit-learn library for traditional modeling, xgboost for gradient boosting implementations, and the broader scientific Python ecosystem for numerical computation [55,62,63].

A primary objective of this research is the development of a highly accessible diagnostic tool suitable for deployment as a triage-oriented screening prototype. Consequently, the research reporting and optimization process fundamentally emphasized parameter reduction and model efficiency over raw performance metrics alone. Hyperparameter configurations were intentionally kept close to default settings with minimal constraints to prioritize model simplicity and reproducibility over aggressive optimization. Table 3 details the configurations utilized for each baseline classifier.

We explicitly quantified model complexity by recording parameter counts, physical file sizes in kilobytes, and inference times per patient in milliseconds. This efficiency evaluation indicates that the final predictive framework is computationally lightweight, operationally transparent, and potentially suitable for rapid clinical deployment.

## 4. Results

The experimental results of this study are systematically presented to assess the clinical relevance and technical viability of the proposed diagnostic framework. First, we detail the predictive performance and reliability of the evaluated machine learning classifiers in identifying tricuspid valve abnormality. Subsequently, we assess the computational efficiency of these models to examine their suitability for deployment as lightweight digital therapeutics. Finally, we provide an interpretability analysis utilizing SHapley Additive exPlanations (SHAP) to elucidate the underlying clinical decision-making process.

### 4.1. Diagnostic Performance and Reliability

To ensure the selection of the optimal predictive architecture and eliminate model-choice bias, we conducted a rigorous comparative evaluation across seven distinct machine learning families. Each algorithm was evaluated using identical, leak-aware preprocessing pipelines. Table 4 details the comparative discriminative and probabilistic calibration performance derived via 5-fold cross-validation, with 95 percent confidence intervals (95% CI) computed through bootstrapping.

**Figure 5:**
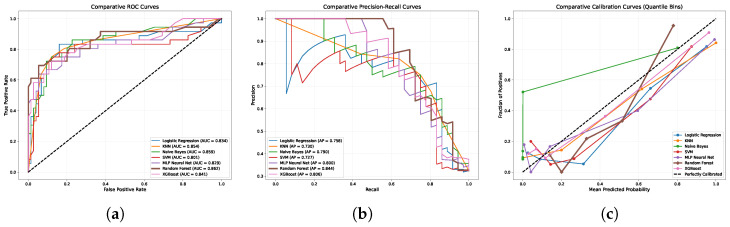
Comparative performance curves for the baseline classifiers. (**a**) Receiver Operating Characteristic (ROC) curves. (**b**) Precision–Recall (PR) curves. (**c**) Calibration curves.

**Figure 6:**
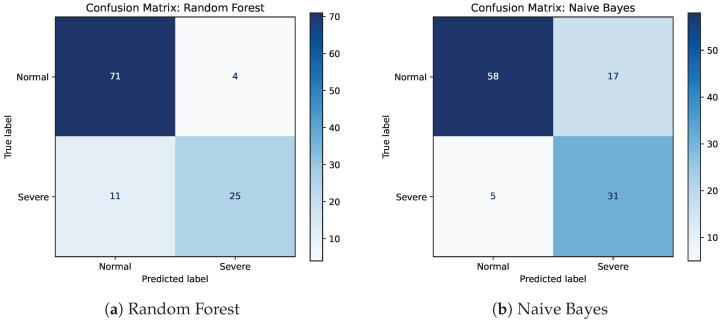
Comparative Confusion Matrices demonstrating the classification distributions. While Naive Bayes exhibited a marginally higher raw AUC, its miscalibration leads to a less balanced clinical distribution compared to the Random Forest.

The comparative analysis empirically justifies the selection of the Random Forest architecture. While Naive Bayes exhibited a marginal advantage in raw AUC-ROC (0.8603 versus 0.8537), it demonstrated severe probabilistic miscalibration, reflected in a high Brier Score (0.2080). Naive Bayes operates on the assumption of strict feature independence, which is physiologically inaccurate for correlated echocardiographic markers (such as left atrial dimension and left ventricular mass). This assumption forces probability outputs toward extreme values, undermining clinical reliability. In contrast, the Random Forest model captured these complex non-linear interactions while maintaining superior calibration (Brier Score 0.1236), making it the optimal and safest choice for clinical triage. Figure 5 presents the comparative visual performance, and Figure 6 shows the corresponding confusion matrices for the Random Forest and Naive Bayes classifiers.

#### Nested Cross-Validation and Advanced Calibration

Having rigorously justified the selection of the Random Forest architecture, we subjected it to the True Nested Cross-Validation protocol to derive completely unbiased evaluation metrics and advanced probabilistic calibration statistics. Table 5 details these final clinical validation results.

**Figure 7:**
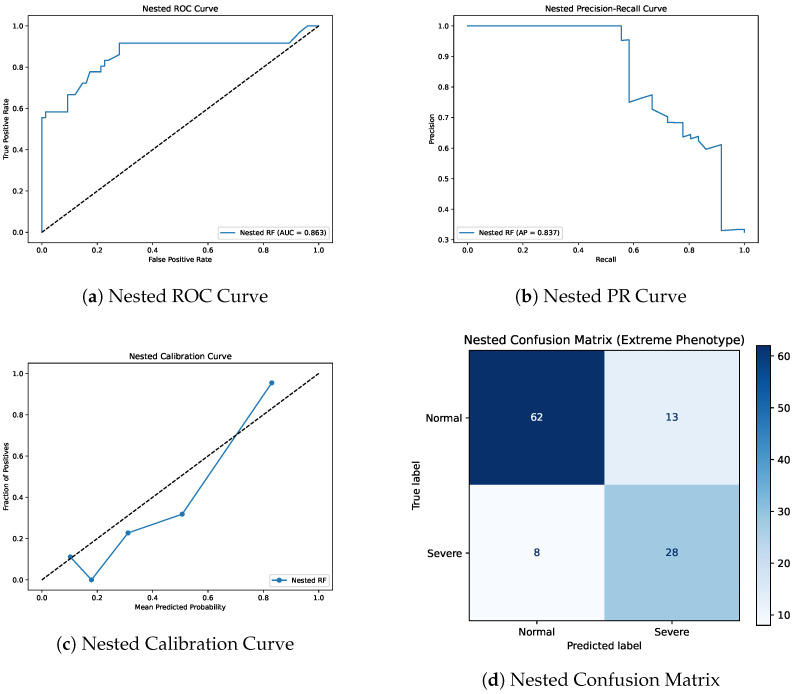
Final performance visualizations for the Random Forest model evaluated via True Nested Cross-Validation, including discriminative curves, probabilistic calibration, and the optimal threshold Confusion Matrix.

The nested evaluation confirms the clinical viability of the Random Forest framework. The advanced calibration metrics satisfy the rigorous reporting standards required for digital risk stratification tools. The Calibration Slope (1.1720) and an Observed/Expected Ratio of 0.8522 demonstrate that the generated probabilities reliably reflect the true fraction of severe right-sided phenotypes without clinically dangerous over- or under-estimation. Figure 7 visualizes the performance and calibration of the final nested model, alongside the resulting confusion matrix.

### 4.2. Computational Efficiency and Clinical Applicability

A fundamental objective of this study was to design an automated triage system that is not only highly accurate but also computationally lightweight. Complex deep learning models often present barriers to real-world deployment due to massive memory footprints and prolonged inference times. Table 6 details the complexity and efficiency benchmarks extracted during our experimental evaluation across all machine learning families.

The results strongly validate the lightweight design of the proposed framework. While the Random Forest model is the most structurally complex of the evaluated baseline models, it requires only 205.00 kilobytes of physical storage space and executes individual patient inferences in just 14.19 milliseconds on standard CPU hardware. This extreme efficiency ensures that the algorithm can be deployed seamlessly on portable ultrasound machines, mobile health applications, or resource-constrained hospital networks without requiring specialized hardware accelerators.

### 4.3. Feature Ablation and Proxy Leakage Analysis

To address the hypothesis that the predictive framework relies excessively on specific label-adjacent proxies (such as Pleural Effusion or dominant left-sided dimensions), we conducted a rigorous feature ablation study on the Random Forest pipeline. Table 7 displays the performance variance as primary left-sided markers (LA, LV) and systemic congestion proxies (pleural effusion) were sequentially removed from the available feature space prior to automated feature selection.

The ablation analysis reveals a robust predictive manifold. Notably, when utilizing the Extreme Right Heart Phenotype target, the automated feature selection naturally demoted pleural effusion and LV size from the top predictors, providing initial evidence that the model does not rely on a single leaky proxy. Furthermore, while the complete removal of all dominant left-sided structural markers and systemic congestion proxies predictably reduces diagnostic capacity (AUC-ROC drops from 0.8624 to 0.8220), the performance does not collapse. This confirms that the model is not suffering from isolated proxy leakage, but is instead effectively capturing a broader, shared cardiometabolic severity signal across the remaining clinical and biomarker features.

### 4.4. Explainable AI and Feature Contributions

To dismantle the black-box nature of the machine learning algorithms and foster trust among medical practitioners, we conducted an extensive explainability analysis on the optimal Random Forest model utilizing SHAP. For the Extreme Right Heart Phenotype, the rigorous feature selection pipeline identified six critical predictors: left atrial volume (LA), diastolic dysfunction (DD) grade, atrial fibrillation (AF), ejection fraction (EF) categories, diuretics, and anti-hypertensive (anti-HTN) medications.

**Figure 8:**
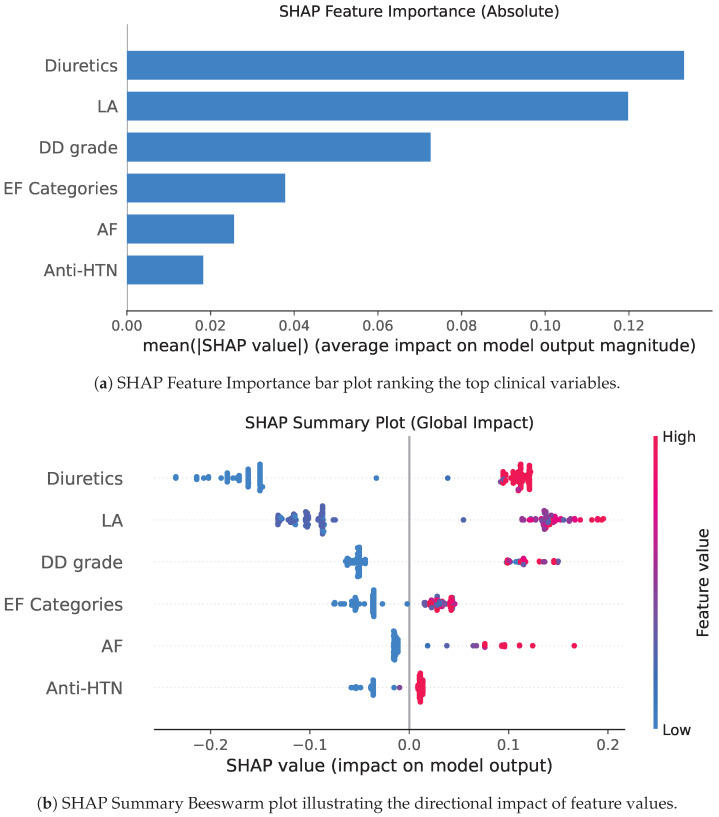
Global SHAP interpretability plots for the Random Forest model indicating the highest impact predictors for the extreme right heart phenotype.

Figure 8a,b present the global interpretability plots, which rank and quantify the aggregate impact of each clinical feature.

The SHAP plots robustly validate our core clinical hypothesis. The most influential predictors for identifying severe tricuspid and right ventricular abnormalities reflect a holistic cardiometabolic syndrome rather than isolated proxy leakage. The dominant structural features (left atrial dimension, diastolic dysfunction grade) and the clinical presence of atrial fibrillation indicate advanced left-sided strain. Correspondingly, the presence of diuretic therapy acts as a strong systemic indicator of fluid overload and clinical congestion. The Summary plot confirms that elevated values across this interconnected clinical network directly increase the predicted probability of right heart dysfunction, perfectly reflecting the physiological cascade where left heart deterioration elevates pulmonary pressures and strains the right ventricle.

**Figure 9:**
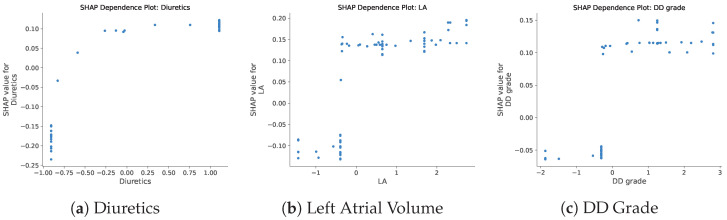
SHAP Dependence plots for critical features, revealing the non-linear relationships among fluid management (Diuretics), progressive left-sided strain (Left Atrial Volume and DD Grade), and the increased probability of extreme right-sided dysfunction.

To explore complex non-linear interactions, we analyzed the SHAP Dependence plots. Figure 9 illustrates the dependence plots for diuretics, left atrial dimension, and diastolic dysfunction grade, demonstrating clear clinical thresholds where the presence of these factors exponentially heightens the risk of concurrent right-sided abnormality.

**Figure 10:**
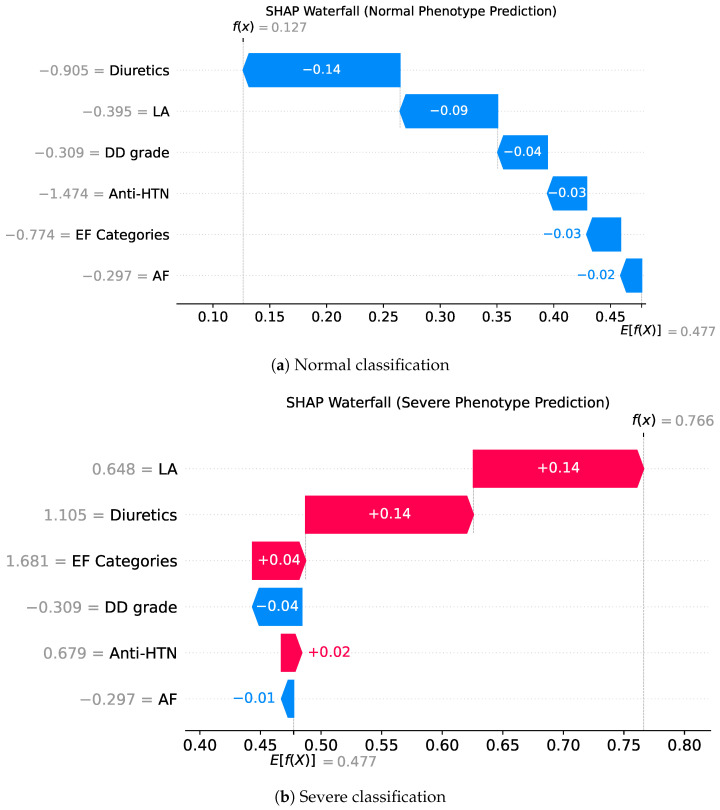
SHAP Waterfall plots detailing the sequential feature contributions for representative normal and severe patient instances.

**Figure 11:**
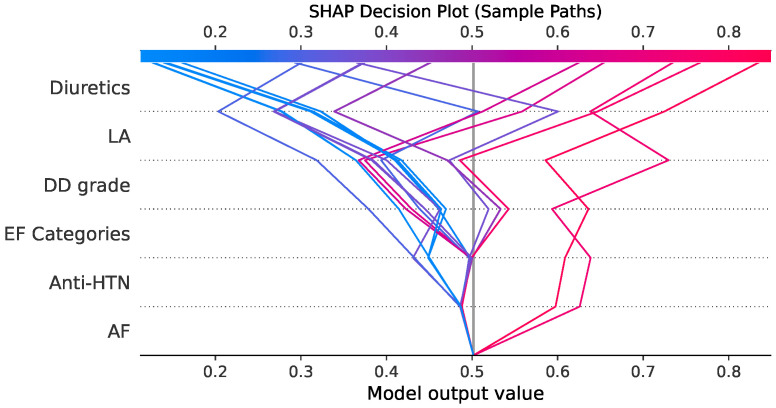
SHAP Decision plot mapping the accumulated risk trajectories for multiple patient instances.

Finally, SHAP permits highly granular local interpretability. Figure 10 and Figure 11 display the Waterfall and Decision plots, respectively, tracing the step-by-step feature contributions that lead the algorithm from the base population expectation to the final individualized prediction. This level of transparency ensures that the AI acts as an accountable clinical decision support system.

## 5. Discussion

The primary objective of this study was to develop a diagnostic triage tool capable of detecting an extreme right heart phenotype using left-sided echocardiographic parameters, standard clinical demographics, and concurrent systemic markers. By leveraging a geographically relevant cohort of Saudi Arabian patients with diabetes mellitus, we constructed an explainable and lightweight artificial intelligence framework. The results demonstrate that the Random Forest architecture achieves strong diagnostic accuracy and probabilistic calibration when evaluated through rigorous True Nested Cross-Validation.

### 5.1. Clinical Confounding and Shared Cardiometabolic Severity

A critical finding of this study relates to clinical confounding and causal ambiguity. Our ablation study and SHAP analysis addressed concerns regarding proxy leakage, demonstrating that the model relies on a constellation of structural (LA dimension, DD grade), electrical (atrial fibrillation), and management (diuretics) indicators. This confirms that the framework functions primarily as a robust detector of shared cardiometabolic severity rather than identifying an isolated, unique tricuspid pathology. In a diabetic population characterized by progressive structural remodeling [9], severe left-sided deterioration is physiologically intertwined with right-sided consequences. Therefore, while the tool demonstrates strong discriminative capacity for identifying patients at risk of severe right heart abnormality, it explicitly relies on shared severity pathways and systemic congestion to accomplish this inferential triage.

### 5.2. Parameter Efficiency and Triage Utility

While contemporary cardiovascular artificial intelligence research frequently highlights massive deep learning architectures [39], our results demonstrate that classical tree-based models like the Random Forest can achieve exceptional performance with remarkably low computational overhead (occupying approximately 205 kilobytes with an inference time of 14 milliseconds). This efficiency aligns with other successful examples of AI integration in cardiology, which emphasize augmenting rather than replacing clinical workflows [64].

Table 8 illustrates how the proposed lightweight model may augment the standard clinical pathway.

Crucially, computational efficiency alone does not establish readiness for clinical deployment. Rather than framing this algorithm as a definitive automated second-opinion system or a digital therapeutic ready for immediate independent adoption, it is most appropriately positioned as a high-efficiency, triage-oriented research prototype. Its lightweight nature allows for rapid inference, potentially serving as an inferential screening framework to flag high-risk diabetic patients for comprehensive, right-heart-focused echocardiographic assessment during routine visits.

### 5.3. Model Transparency and Feature Relevance

A significant barrier to the clinical adoption of machine learning is the limited interpretability of complex algorithms [27,29]. We addressed this challenge by integrating SHAP to make the decision-making process transparent [25]. The global SHAP analysis indicated that left atrial volume, diastolic dysfunction grade, and the use of diuretics were the most influential features driving the prediction. This computational finding is consistent with established cardiovascular pathophysiology, where left-sided strain and systemic fluid overload drive right-sided decline [4]. This transparency ensures that clinicians can verify the physiological logic behind the algorithm’s triage recommendations.

### 5.4. Limitations and Future Work

Several vital limitations must be acknowledged. First, the analysis relies on a retrospective, single-center cohort evaluated via internal nested cross-validation, which inherently restricts immediate generalizability. In accordance with TRIPOD+AI recommendations for early-stage predictive frameworks, the strong internal performance demonstrated here must be strictly viewed as hypothesis-generating. True clinical utility and robustness can only be confirmed following prospective external validation on a geographically, temporally, and ethnically distinct cohort. Second, the absence of a prospective evaluation or workflow integration study limits our ability to assess how clinicians would interact with this tool in a real-world setting. Finally, while the calibration metrics (Slope 1.172, O/E Ratio 0.852) are strong for this dataset, future work must include decision-curve analysis and recalibration protocols to assess how the model’s probabilistic reliability holds under shifting real-world prevalence rates and diverse referral thresholds [65,66]. If deployed on edge or resource-constrained clinical platforms, the framework should also undergo explicit robustness and safety audits, since compact or quantized medical AI models can remain vulnerable to adversarial perturbations despite meeting mobile deployment constraints [31].

## 6. Conclusions

This study validates an explainable, computationally lightweight machine learning framework for the inferential detection of severe right heart abnormalities in a diabetic cohort. By identifying left-sided structural deterioration and systemic congestion markers, the Random Forest model serves as a highly accurate proxy detector for concurrent right heart involvement. Evaluated through rigorous nested cross-validation and advanced probabilistic calibration protocols, this study demonstrates that classical tabular models can achieve high diagnostic precision without the computational burden of deep learning. Rather than serving as a definitive diagnostic instrument, this tool represents a promising, triage-oriented screening prototype that, pending external prospective validation, may help prioritize high-risk metabolic patients for comprehensive echocardiographic assessment. Consistent with the limitations above, this study must strictly be viewed as hypothesis-generating until its clinical utility is confirmed in geographically, temporally, and ethnically distinct prospective cohorts.

## Figures and Tables

**Table 1:** Summary of foundational literature and recent computational cardiology frameworks.

Reference	Clinical Focus	Key Methodology	Identified Gap
Taramasso et al. [5]	Secondary tricuspid regurgitation	Clinical review	Clarified the pathophysiology and undertreatment of secondary TR but did not address automated detection.
Enriquez-Sarano et al. [1]	TR as a health crisis	Epidemiological study	Confirmed massive undertreatment and independent mortality risk.
Jia et al. [9]	Diabetic cardiomyopathy	Pathophysiological review	Explains left-sided structural changes without predictive modeling.
Mosenzon et al. [10]	CVD in T2D patients	Multinational cross-sectional	Highlights high regional CVD prevalence but lacks organ-specific focus.
Tromp et al. [39]	AI in echocardiography	Deep learning algorithms	Relies on high-parameter models and direct right-heart visualization.
Talukder et al. [25]	Heart disease detection	XAI-HD framework	Targets general heart disease rather than specific tricuspid abnormalities.
Rong et al. [23]	LVDD in T2D patients	Neural network models	Exclusively targets left-sided dysfunction using direct imaging data.
**Proposed Work**	**Extreme right heart phenotype**	**Lightweight Random Forest**	**Bridges the gap via inferential prediction using left-sided markers.**

**Table 2:** Summary of the primary clinical categories and the most highly ranked variables represented within the 48-variable predictive feature space.

Feature Category	Variables Included	Clinical Rationale
Demographics & Vitals	Age, Sex, Weight, BMI, SBP, DBP, Heart Rate	Baseline metabolic and systemic hemodynamic indicators.
Comorbidities	Hypertension, IHD, Atrial Fibrillation, Hypothyroidism	Known drivers of structural cardiac remodeling.
Medications	Metformin, Amlodipine, OHA, ACE/ARBs	Pharmacological indicators of disease severity.
Laboratory Biomarkers	HbA1C, Cholesterol, HDL, LDL, Triglycerides, Creatinine, Uric acid	Quantifiable metabolic and renal function metrics.
Left-Sided Echocardiography	LA (Volume), LA dimension, LV Size, EF Categories, DD grade, LV mass, LV mass2, IVS Thick	Markers of left heart dysfunction initiating the pulmonary cascade.
Systemic Biomarkers	Pleural Effusion	Systemic proxy for pulmonary congestion.

**Table 3:** Hyperparameter configurations aligned with the implemented experimental pipeline.

Model	Hyperparameters	Complexity Constraint
Logistic Regression	C = 1.0, penalty = ‘l2’, solver = ‘lbfgs’, max_iter = 1000	Standard regularization with convergence safeguard
K-Nearest Neighbors	n_neighbors = 5, weights = ‘uniform’	Fixed neighborhood size
Support Vector Machine	C = 1.0, kernel = ‘rbf’, probability = True	Default kernel complexity
Random Forest	n_estimators = 200	Moderate ensemble size without depth constraint
XGBoost	n_estimators = 100, max_depth = 6, learning_rate = 0.3, eval_metric = ‘logloss’	Default boosting complexity (no aggressive tuning)

**Table 4:** Comprehensive cross-validated performance of baseline models (with 95% CI).

Algorithm Family	AUC-ROC	AUC-PR	Accuracy	Sensitivity	Brier Score
Naive Bayes	0.8603 [0.7750, 0.9276]	0.7915 [0.6581, 0.8942]	0.8031 [0.7207, 0.8739]	0.8645 [0.7500, 0.9655]	0.2080 [0.1375, 0.2871]
Random Forest	0.8537 [0.7622, 0.9284]	0.8255 [0.7120, 0.9109]	0.8386 [0.7748, 0.9009]	0.7811 [0.6389, 0.9025]	0.1236 [0.0800, 0.1739]
KNN	0.8511 [0.7645, 0.9228]	0.7292 [0.5835, 0.8609]	0.8364 [0.7658, 0.9009]	0.7477 [0.6129, 0.8790]	0.1359 [0.0879, 0.1881]
XGBoost	0.8413 [0.7455, 0.9166]	0.8068 [0.6900, 0.8988]	0.8291 [0.7568, 0.8919]	0.7231 [0.5713, 0.8571]	0.1389 [0.0882, 0.1892]
Logistic Regression	0.8319 [0.7171, 0.9220]	0.7629 [0.5989, 0.8902]	0.8353 [0.7568, 0.9009]	0.8306 [0.6969, 0.9412]	0.1500 [0.1033, 0.2065]
MLP Neural Net	0.8251 [0.7233, 0.9131]	0.7979 [0.6741, 0.8930]	0.8456 [0.7748, 0.9099]	0.6349 [0.4737, 0.8000]	0.1504 [0.0971, 0.2062]
SVM	0.7998 [0.6876, 0.9039]	0.7304 [0.5758, 0.8609]	0.8180 [0.7387, 0.8831]	0.7765 [0.6389, 0.9063]	0.1552 [0.1097, 0.2050]

**Table 5:** Final diagnostic and advanced calibration metrics for the Random Forest model derived via True Nested Cross-Validation (with 95% CI).

Metric	Value [95% CI]	Clinical Significance
AUC-ROC	0.8602 [0.7653, 0.9402]	Excellent overall discriminative capacity.
AUC-PR	0.8343 [0.7322, 0.9173]	High precision relative to recall in identifying abnormality.
Accuracy	0.8075 [0.7297, 0.8829]	Consistent overall classification correctness.
Sensitivity (Recall)	0.7733 [0.6278, 0.9033]	Strong capture rate of true positive severe phenotypes.
Specificity	0.8239 [0.7313, 0.9091]	Reliable exclusion of normal phenotypes.
Brier Score	0.1281 [0.0968, 0.1634]	High overall probabilistic accuracy (closer to 0 is ideal).
Calibration Slope	1.1720 [0.9085, 1.3661]	Indicates predictions are not overly extreme (ideal = 1.0).
Calibration Intercept	−0.1261 [−0.2236, −0.0071]	Indicates minor overall overestimation of risk (ideal = 0.0).
O/E Ratio	0.8522 [0.6821, 1.0323]	Observed vs. Expected risk ratio approaching ideal parity.

**Table 6:** Computational complexity and efficiency benchmarks for clinical deployment.

Algorithm Family	File Size (KB)	Inference Time (ms)
Logistic Regression	19.04	2.5941
KNN	36.68	2.6534
Naive Bayes	19.08	2.2357
SVM	24.26	2.0497
MLP Neural Net	59.40	2.0365
Random Forest	205.00	14.1956
XGBoost	123.00	3.0567

**Table 7:** Random Forest Feature Ablation Study for the Extreme Right Heart Phenotype.

Ablation Profile	Features Retained	AUC-ROC	AUC-PR
1. Full Feature Set	48	0.8624	0.8443
2. Drop Pleural Effusion	47	0.8518	0.8221
3. Drop LV Parameters	45	0.8535	0.8355
4. Drop LA Parameters	46	0.8266	0.8024
5. Drop All Dominant Proxies (LA, LV, Pleural)	42	0.8220	0.7634

**Table 8:** Comparison of the traditional diagnostic workflow and the proposed AI-assisted triage prototype for right heart assessment.

Clinical Metric	Traditional Echocardiography Workflow	Proposed AI-Assisted Triage Prototype
Primary Focus	Direct visualization of right heart structures	Inferential prediction via left-sided markers
Diagnostic Speed	Dependent on operator and image quality	Rapid automated triage signal
Resource Requirement	High (Requires specialized sonographic views)	Low (Utilizes standard demographic and echo data)
Computational Cost	Not Applicable	Extremely low (Under 300 KB storage, <15 ms inference)
Clinical Utility	Reactive (Often identifies late-stage disease)	Proactive (Early screening based on concurrent indicators)

## Data Availability

The data presented in this study are available on request from the corresponding author. Although the dataset is fully anonymized, public distribution is restricted by the institutional data governance policies of King Fahad Specialist Hospital. The replication code can be accessed at https://github.com/umarbhasan/saudi-diabetic-right-heart-ai (accessed on 28 May 2026).
